# Mobilisation of data to stakeholder communities. Bridging the research-practice gap using a commercial shellfish species model

**DOI:** 10.1371/journal.pone.0238446

**Published:** 2020-09-23

**Authors:** Kate E. Mahony, Sharon A. Lynch, Sian Egerton, Sara Cabral, Xavier de Montaudouin, Alice Fitch, Luísa Magalhães, Mélanie Rocroy, Sarah C. Culloty

**Affiliations:** 1 School of Biological, Earth and Environmental Sciences, and Aquaculture and Fisheries Development Centre (AFDC), and MaREI Centre, Environmental Research Institute (ERI), University College Cork, Cork, Ireland; 2 MARE–Centro de Ciências do Mar e do Ambiente, Faculdade de Ciências, Universidade de Lisboa, Campo Grande, Lisboa, Portugal; 3 Université de Bordeaux, CNRS, UMR 5805 EPOC, Station Marine d’Arcachon, Arcachon, France; 4 UK Centre for Ecology & Hydrology, Environment Centre Wales, Bangor, United Kingdom; 5 Departamento de Biologia & CESAM, Universidade de Aveiro, Aveiro, Portugal; 6 GEMEL Groupe d'Étude des Milieux Estuariens et Littoraux, Saint-Valery-sur-Somme, France; Texas A&M University, UNITED STATES

## Abstract

Knowledge mobilisation is required to “bridge the gap” between research, policy and practice. This activity is dependent on the amount, richness and quality of the data published. To understand the impact of a changing climate on commercial species, stakeholder communities require better knowledge of their past and current situations. The common cockle (*Cerastoderma edule*) is an excellent model species for this type of analysis, as it is well-studied due to its cultural, commercial and ecological significance in west Europe. Recently, *C*. *edule* harvests have decreased, coinciding with frequent mass mortalities, due to factors such as a changing climate and diseases. In this study, macro and micro level marine historical ecology techniques were used to create datasets on topics including: cockle abundance, spawning duration and harvest levels, as well as the ecological factors impacting those cockle populations. These data were correlated with changing climate and the Atlantic Multidecadal Oscillation (AMO) index to assess if they are drivers of cockle abundance and harvesting. The analyses identified the key stakeholder communities involved in cockle research and data acquisition. It highlighted that data collection was sporadic and lacking in cross-national/stakeholder community coordination. A major finding was that local variability in cockle populations is influenced by biotic (parasites) and abiotic (temperature, legislation and harvesting) factors, and at a global scale by climate (AMO Index). This comprehensive study provided an insight into the European cockle fishery but also highlights the need to identify the type of data required, the importance of standardised monitoring, and dissemination efforts, taking into account the knowledge, source, and audience. These factors are key elements that will be highly beneficial not only to the cockle stakeholder communities but to other commercial species.

## 1. Introduction

Knowledge does not transmit well [1], resulting in a gap between research and end users, with information not always translating from researchers to policy makers, the public and resource managers [2]. This has led to the study of knowledge mobilisation, which relates to the flow and application of information [3]. Such transfer of information improves outcomes in sectors such as health [4], education [5] and conservation [6]. However, obstacles to knowledge mobilisation and exchange exist, including difficulties such as secrecy of data by certain institutions [1], lack of open data [7] and a shortage of collaboration between all relevant stakeholder groups [3]. Such sharing of information should be intuitively easier on a well studied topic, due to an abundance of literature.

The common cockle (*Cerastoderma edule*, Mollusca: Bivalvia: Cardiidae) is an extensively studied species. Its populations have a large geographic range, being found along European Atlantic coasts from Norway to Senegal [8,9]. Cockles provide a wide range of services, including cultural and ecosystem services [10]. As an ecosystem engineer, it alters its habitat by bioturbation and influencing hydrodynamics [11,12]. Additionally, cockles are a valuable species to European fisheries, with capture production reaching over 100,000 tonnes per year in the 1980s and early 1990s. However, in similarity with other fisheries [13], the production of cockles has since reduced [14], coinciding with reports of changing fisheries policy, significant overfishing, variable recruitment and mass mortalities [15]. Some of these reported mortalities are a result of climate related events (e.g. high precipitation, storms and heat waves), which are anticipated to increase in frequency over the coming decades [16–18]. The cockle is also impacted on by a wide range of parasites and pathogens [19–21]. For instance, the recent discovery of the pathogen *Marteilia cochillia* is causing significant mortalities in Galicia, Spain [22].

The ecological niche of *C*. *edule* is predicted to narrow in response to climate change [23] and its range of distribution may shift northwards in response to increasing temperatures [24]. Global trends in cockle densities have been observed previously, such as densities increasing towards higher latitudes [25]. Decreases in abundance have also been documented at a regional level, for example in the Wadden Sea in the Netherlands [26], possibly due to overfishing and eutrophication [27]. Changes in reproduction of cockles have been observed at regional levels as a result of climate variability, with cold winters causing an acceleration of gametogenesis, as well as extending its duration [28]. At an estuary scale, in southern Portugal overfishing of cockles has resulted in disruptions to population structure due to the removal of larger individuals [29]. This highlights the importance of examining cockles temporally at a metapopulation level, as well as across their distribution in a single comprehensive study that can be communicated effectively to relevant stakeholders.

In order to understand the ecology and status of current and future marine populations, including cockles, an understanding of past events, such as mortality events, changes in population structure and dynamics, as well as health and disease related impacts, is required. Marine historical ecology “is an emerging field of study that uses historical data sets to describe what marine ecosystems might have looked like in the past" [30]. Pauly [31] raised the issue of the "shifting baseline syndrome" in which fishery scientists focus and generate data from their own career span to determine changes and baseline shifts across generations [31]. However, to get a longer, less biased perspective of marine resource status, it is important to familiarise oneself with the data and mobilise knowledge from a wide range of archival sources, including natural sources such as fossils and documentary records, including maps [32,33]. These techniques have been successfully incorporated into previous studies on commercially important species. In a study directed at the scientific community, fishers’ logbooks were used as a data source for cod biomass to understand the health of stocks prior to the industrialisation of the fishery [34]. With 64 citations it is valid to say that this study had substantial impact on the scientific audience. Historical studies have also been conducted on bivalve species, primarily oysters, to understand their historic distribution and abundance [35], as well as to examine potential causes for declines, such as disease and over harvesting [36].

In this study, a review was conducted of the available historical information on a commercially important species, the common cockle. Using this review, the aim was to gain an understanding of (1) the regional and national focus of past studies (“the knowledge”), (2) the sources of knowledge available on cockles, (3) the vested stakeholder communities (“audience”) and (4) to identify some of the key drivers and inhibitors of cockle populations—both on a macro and micro level (“knowledge mobilisation”). Historical data are inherently fragmented and variable in size and quality, but with data cleaning techniques, these data can provide invaluable information on some of the previous factors that have influenced cockle population growth or decline. Factors such as differences in legislation, fishing intensity, level of protection, minimum landing size, disease, reproduction and environmental variables were examined in this study. Based on these differences, case studies (micro level) of three key production sites were also examined, to determine if trends varied at a local scale. Data was also analysed on a macro level to examine the impacts of climate on cockle populations. Findings from this study will contribute to knowledge mobilisation for a commercially and ecologically important species and will further reduce the research-practice gap in this sector.

## 2. Materials and methods

### 2.1. Rationale

To understand “the knowledge”, i.e. the knowledge of cockles from past studies at both a regional and global scale, a review of secondary data (obtained from historic literature reporting original data) was conducted, as described in detail in Section 2.2. As this data was gathered for other purposes (e.g. to inform industry, for publication) it was deemed important to understand “audience”, i.e. stakeholders this knowledge was intended for, as well as the stakeholder community providing the data, i.e. “the source”. These sources provide records of cockle populations, which were examined on a macro and micro level to depict trends, as well as the efficiency of “knowledge mobilisation” in this sector.

### 2.2. Literature review, data set creation and standardisation

Data sets detailing density, biomass, spatial distribution, spawning duration and landing records were created by carrying out a web-based literature search. Searches of both grey and published literature were conducted through Google Scholar, using the synonyms “*Cardium edule*” or “*Cerastoderma edule*”, as well as through individual search tools (ScienceDirect, JSTOR and Wiley). Literature was accessed to 2018 inclusive. Authors’ personal libraries were also searched for relevant literature. Not all of this data was readily accessible on the internet due to pay walls, lack of digitisation and language barriers. It is important to set explicit criteria when creating data sets from multiple studies [37]. In the case of this study, key inclusion criteria were as follows: biomass and density minimum and maximum, biomass and density estimates, parasite records, spawning period, capture production, and presence data from museum records and newspapers. Efforts were taken to ensure data was clean, with studies excluded if they were experimental, lacked a stated time period or location or if the area that biomass was measured over was not clearly indicated. Each individual quantitative measure was referred to as a record, e.g. if two time periods were recorded in one piece of literature, this counted as two records. Care was taken to omit studies focussing on the lagoon cockle (*Cerastoderma glaucum*), also present in Europe, and studies were only included if *C*. *edule* was specifically stated.

**Fig 1:**
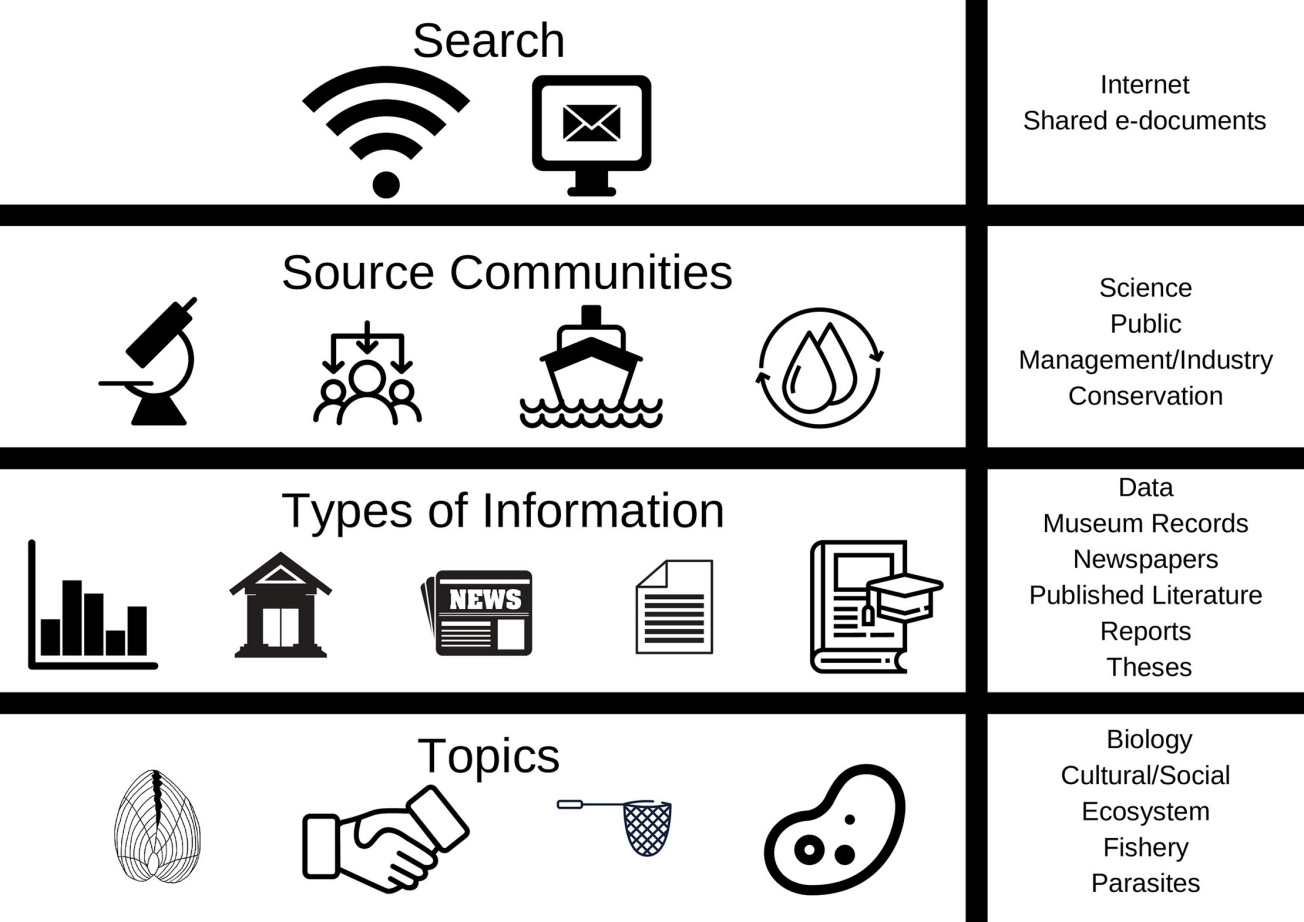
Overview of the data types, sources and topics collated to facilitate knowledge mobilisation of common cockle *Cerastoderma edule* research to key stakeholder communities. This figure illustrates the methodology used and provides a template for carrying out such a study.

Studies were classed according to the “stakeholder community” from which they originated (Fig 1). Literatures sourced from the “Science” community originated in the published literature or from scientific reports. The “Management/Industry” community was assigned if the literature originated from a body overseeing a fishery. Knowledge was classed as “Public” if it originated from public media and “Conservation” was assigned in cases where literature related to conservation objectives, such as protected areas. Literature was further classified according to its topic, e.g. ecosystems, fisheries, parasites (Fig 1, S1 Table). No ambiguous cases were noted, i.e. all studies were easily assigned to a community.

Additional data cleaning was conducted prior to interpretation, where abundance data (biomass and density) were converted and standardised, so all figures were reported as the same unit (g wet weight/m^2^ and individuals/m^2^ respectively). Biomass was reported in many ways in the literature. The principal units were ash free dry weight (AFDW) and wet weight. All biomass data were converted to wet weight. AFDW was converted using the following equation from Wijsman, Brummelhuis, & Smaal [38]:

$$WW=\frac{AFDW}{{\phi AFDW}_{\_ww}}$$

where *WW* = wet weight, *AFDW* = ash free dry weight and, *ϕAFDW*_*_WW*_ = conversion factor.

A conversion factor of 0.12 was used to convert ash free dry weight to wet weight [39].

For the climate analyses, Atlantic Multidecadal Oscillation (AMO) data, ranging from 1956 to 2018 were obtained [40]. The AMO is a cycle of variability of Atlantic climate over an extended timescale. A negative AMO index corresponds with a cooler Atlantic sea surface temperature, conversely a positive AMO corresponds with a warmer Atlantic sea surface temperature [41]. These data were averaged yearly to determine the annual AMO Index. Precipitation and maximum and minimum average temperatures were obtained from national weather forecasts, from nearest weather stations [42–44].

### 2.3. Analysis

Linear mixed effect models fit by restricted maximum likelihood were employed to investigate fluctuations in cockle densities. Models were generated in R [45] using the lme function in the nlme package [46]. The fixed factors included were AMO index, season, year, cockle age, sampling type and latitude (S2 Table) and the random factors were year and latitude (in order to account for the spatial and temporal variation in the reports). The final model was chosen according to top down selection [47], using a combination of AIC and *p* values to determine the most suitable model, and subsequently removing the over-specified fixed factors (S3 Table). Homoscedascity (homogenous variance of residuals) and normality (normally distributed residuals) assumptions were visually checked (S1 Fig).

Due to the variation of sampling techniques in the reported/detected cockle studies, as well as the infrequent reporting of density and biomass on a global scale, it was impossible to conduct a statistical analysis for the remainder of the study. Instead, data were analysed descriptively to determine potential trends across the distribution of the common cockle. Case studies (micro level) were also conducted on a regional scale (Dundalk, Ireland; Bay of Somme, France; Ria de Arousa, Spain) to determine the climate related factors influencing cockles. These sites were chosen from three latitudinal areas based on the amount of data available.

## 3. Results

### 3.1. Overview of results and sources

The total number of records obtained was 9,997 from 193 sources (e.g. museum records, published literature, reports etc.). Of these records, 2,814 were quantitative, detailing the abundance of cockles, harvest amounts or spawning duration. A total of 7,183 records were presence data, which were used in combination with the quantitative data to determine the spatial distribution of cockles. The oldest record of cockles was from a museum specimen from Lough Hyne, Ireland in 1859, the most recent record included was from 2018 harvest data in Galicia, Spain. Museum and newspaper records were only obtained from Irish sources, with the majority (7,105) of these records from Ireland (294) and the UK (6,811). The Irish sources also documented records from other countries, with remainder of these records obtained from Spain (43), France (29) and Portugal (6).

**Fig 2:**
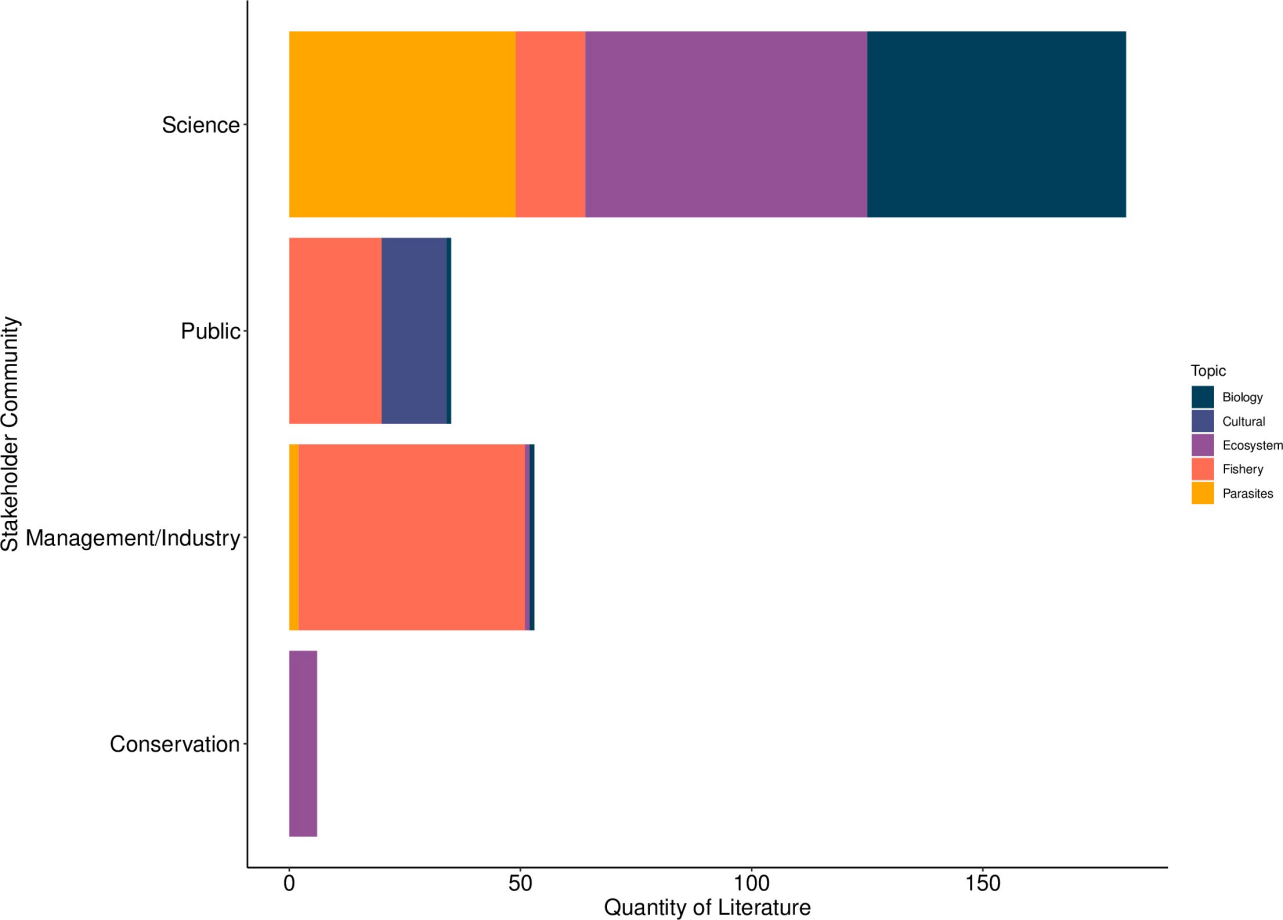
Stakeholder communities providing literature for this study, including the topic of the literature/dataset. Literature relating to cockles covered the areas of cockle biology, reproduction and distribution. The topic of “Biology” refers to the biology and natural history of cockles. The topic of “Culture” dealt with the relationship between people and cockles (e.g. gastronomy). “Ecology” related to the interaction between cockles and their environment (e.g. sediment) and other species (e.g. predators). The topic “Fishery” related to cockle harvest levels and “Parasites” was classified as literature reporting parasites in cockles.

The majority of literature provided was obtained from the scientific community (Fig 2), covering a large range of topics. The least amount of literature was provided in the context of conservation. Management and industry provided literature regarding the fishery, primarily reporting landings statistics. However, small quantities of literature sourced from management and industry related to cockle biology and their parasites. No qualitative information was provided from the public realm, as fisheries related literature primarily discussed the dangers of cockle fishing, or simply the presence of a fishery in a specific area.

### 3.2. Cockle and stakeholder community distribution

**Fig 3:**
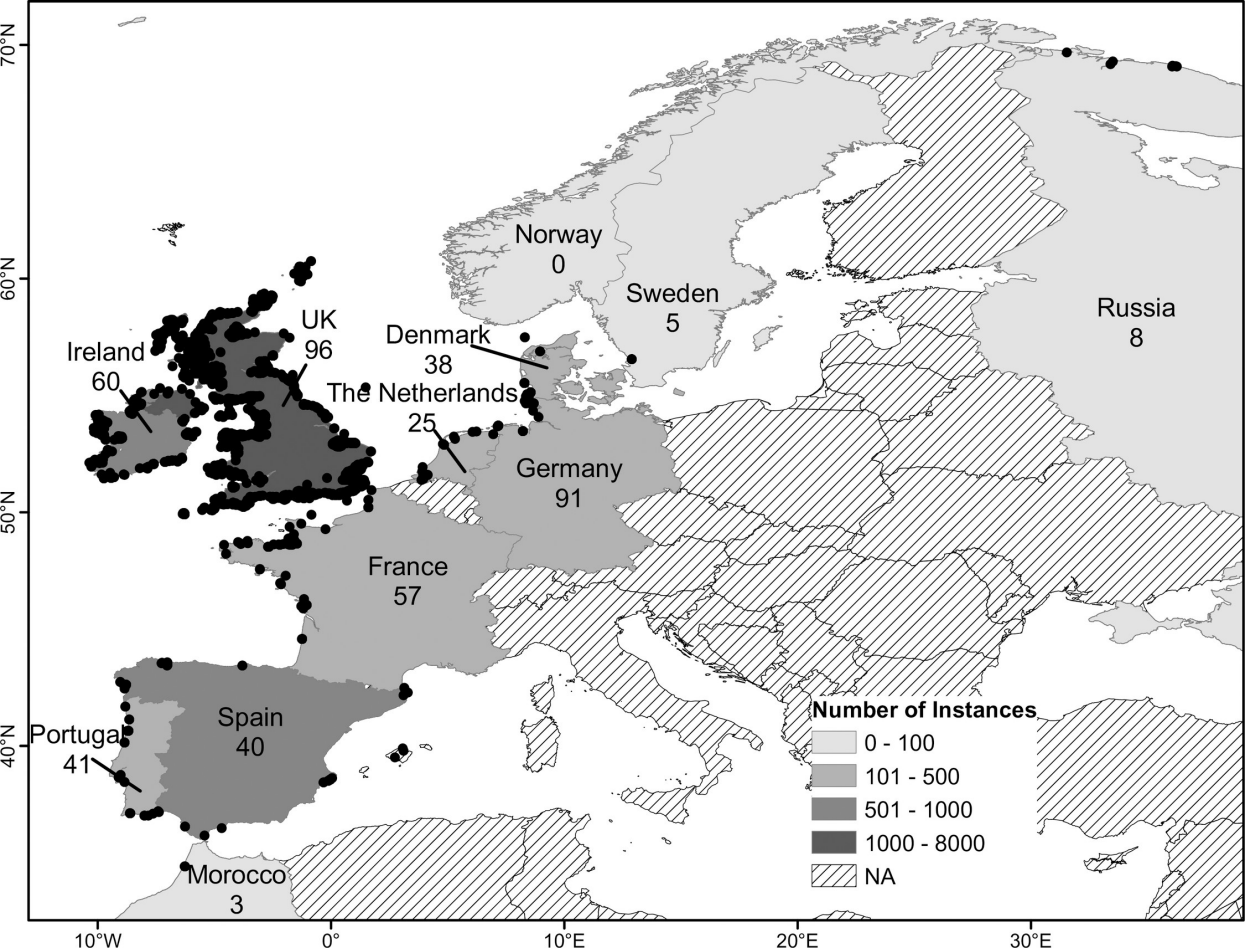
European distribution of cockles from all data gathered regarding *Cerastoderma edule*. The total number of reports (presence, production, abundance) are indicated by grey colour as seen in the legend. The number of reports including a quantitative measure of abundance (i.e. density or biomass) are represented by text within the map. Note: records were obtained from Iceland, southern Morocco, Mauritania and Senegal but omitted for clarity. Made with Natural Earth. Free vector and raster map data @ naturalearthdata.com.

All historical records were used to determine the documented distribution of *C*. *edule* (Fig 3), which also highlighted the regional/local geographic range of stakeholder communities/studies carried out. Cockles were recorded as far as Russia [48,49] and Iceland [50] in the north, to Senegal in the south [14]. *C*. *edule* was reported to be found on the Mediterranean coasts of Spain [51]. Whereas no reports of cockles came from the Mediterranean coasts of France, Italy or further eastern areas (Fig 3). No records were observed over a large distance between Morocco and Senegal, with the exception of one study [9].

### 3.3. Historical abundance (macro level analysis)

**Fig 4:**
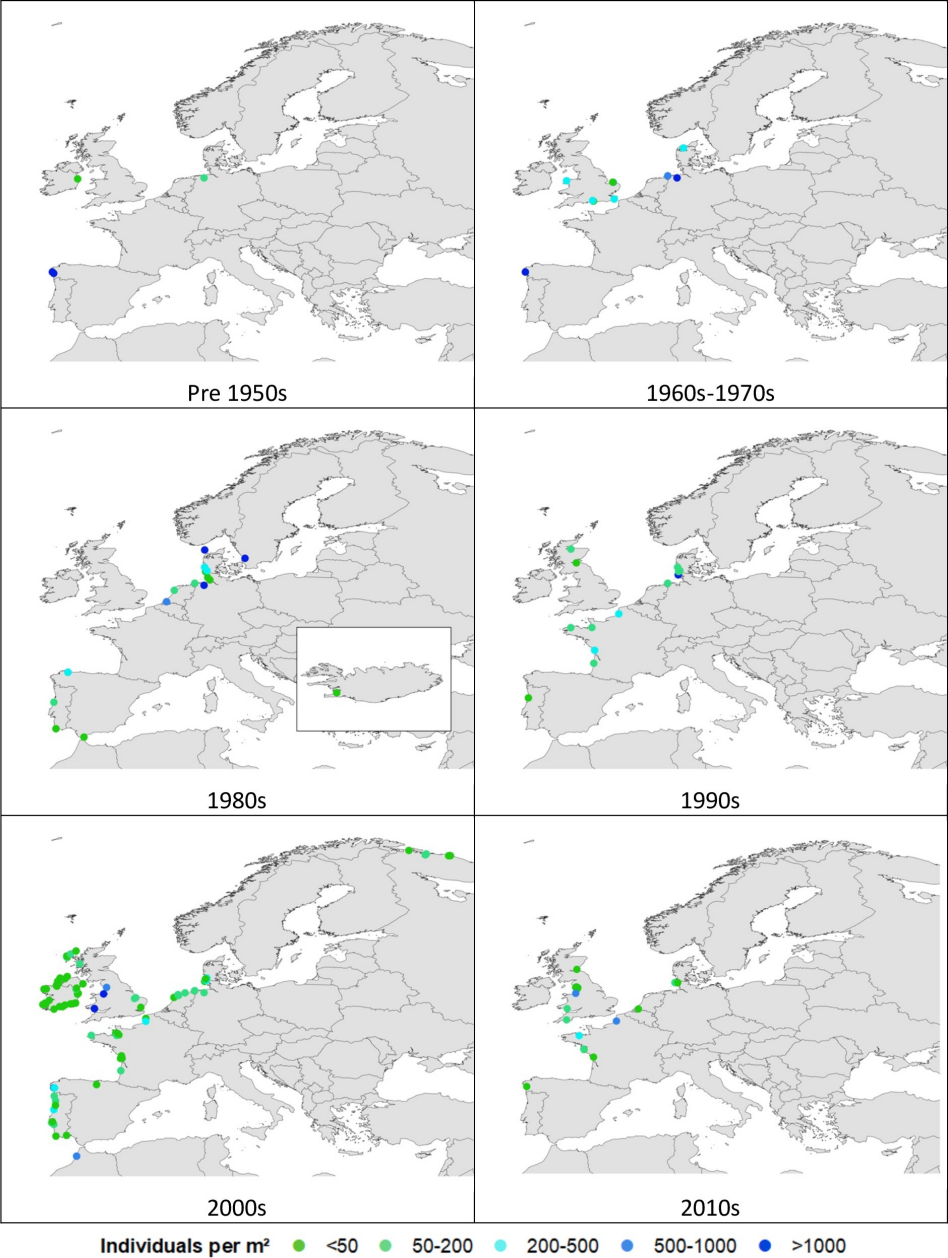
Density of cockles reported across Europe between the 1940s and 2010s. The colours of dots indicate the number of individuals per m^2^. Made with Natural Earth. Free vector and raster map data @ naturalearthdata.com.

Abundance (i.e. the number or weight of cockles per m^2^) was recorded as either biomass or density and varied spatially and temporally in the analysed studies. The south coast of Ireland and the Wadden Sea were amongst the most frequently studied areas (Fig 4). The countries with the most records of cockle abundance were the UK (96 records), followed by the Germany (91 records) (Fig 3). Ireland (60), France (57), Portugal (41), Spain (40), Denmark (38) and the Netherlands (25) also had a significant number of reports on cockle abundance (Fig 4). Countries with small/no cockle fisheries had the lowest numbers of reports e.g. Russia (8), Iceland (1) and Senegal (0) (Fig 4).

Records of biomass (g Wet Weight/m^2^) varied spatially and temporally. The earliest biomass record (185 gWW/m^2^) was from Dublin Bay, Ireland, in 1971 [52]. Few records exist from the 1970s, while many surveys detailed biomass in the 2000s. The south coast of Ireland and the Wadden Sea were amongst the most frequently studied areas, with no records from the south of Portugal. Biomass dramatically varied in certain locations, even within the same decade. For example, in the east of Ireland, where biomass ranged from 134 gWW/m^2^ in 2008 to 30.59 gWW/m^2^ in 2010 in a single bay [53], or in Arcachon Bay, France, where values ranged from 0 gWW/m^2^ to 460 gWW/m^2^ between 1997 and 2014, with no temporal trend [54]. Similarly, biomass in Wales was higher in the 2000s than the 1990s. The highest recorded biomass was in the UK, reaching 2,873.1 gWW/m^2^ in the Burry Inlet in 2009 [55].

Examining the density of cockles by decade, most areas appear to have similar cockle densities temporally. However, spikes in reported densities appeared in the 1980s in the Wadden Sea and in the 2010s in the south of the UK. Density appeared lower in areas at the north of the cockles’ range, including Ireland, northern UK, Iceland and Russia. The highest reported densities were in key fishing areas in the UK, the Wadden Sea, Galicia and southern Portugal (Fig 4).

**Fig 5:**
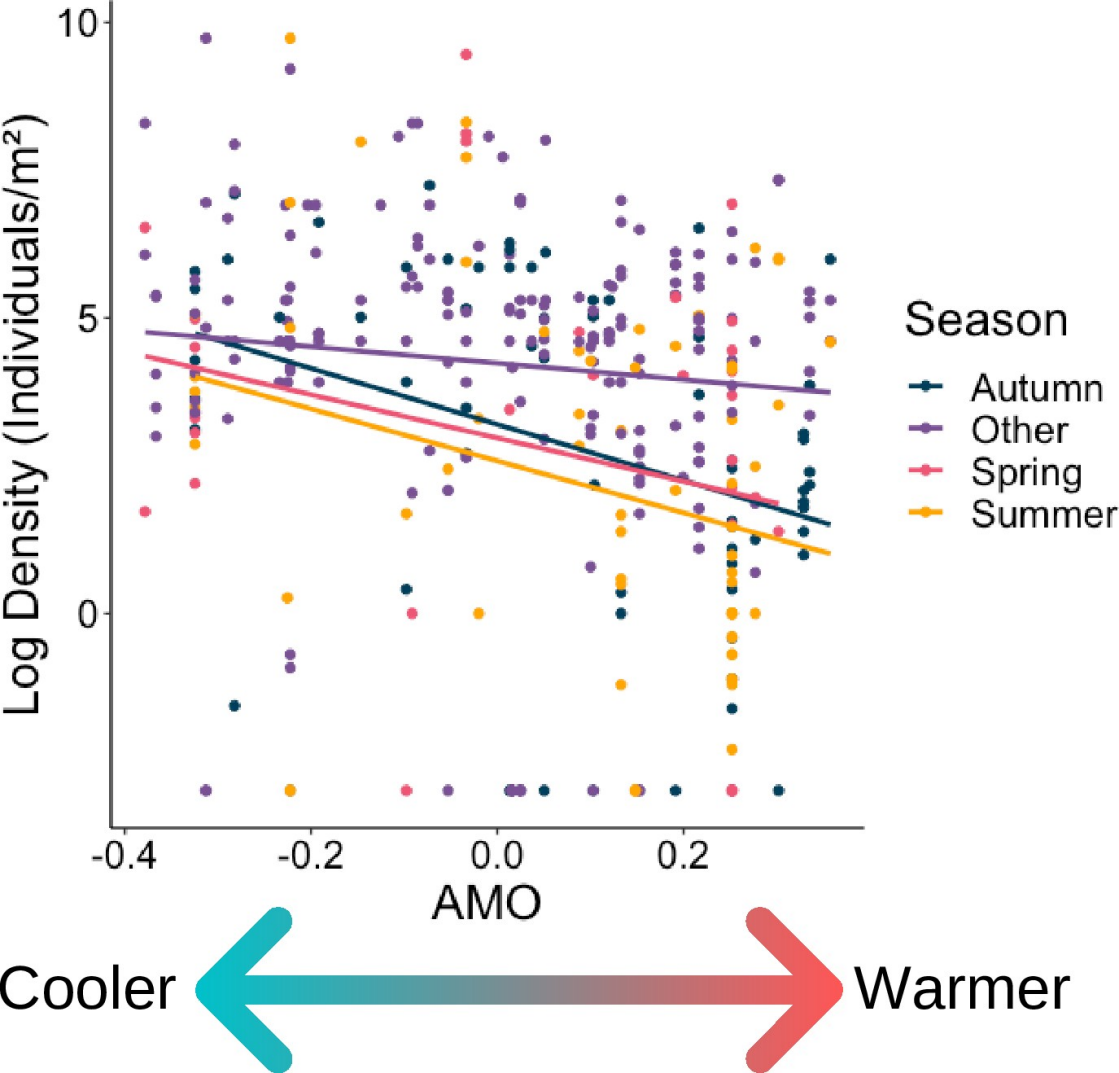
Relationship between log_10_ density of cockles (*Cerastoderma edule*) and the AMO index. The AMO index and sampling season had a significant impact on cockle density.

**Fig 6:**
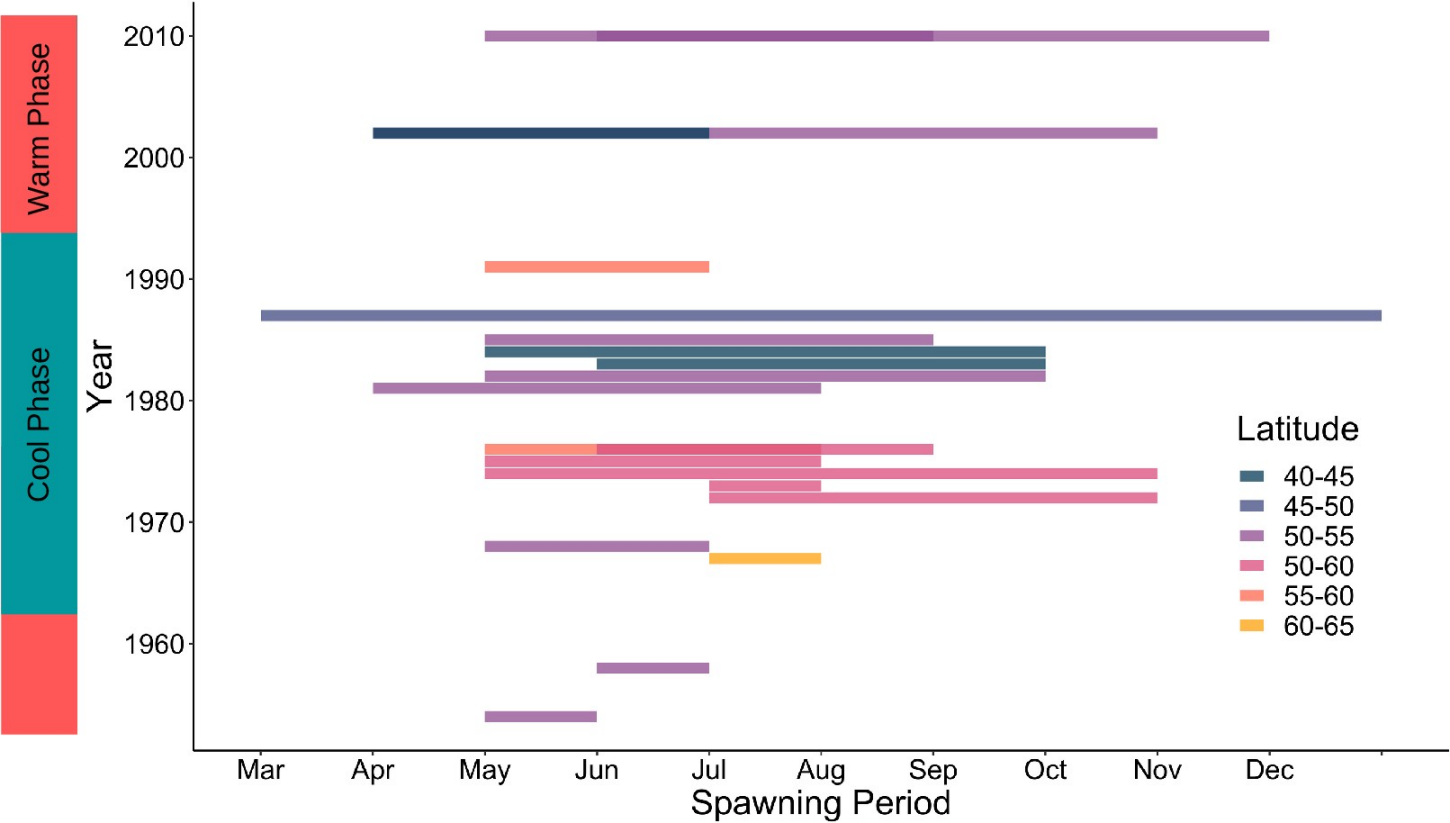
Historic spawning period of *Cerastoderma edule*, across various latitudes in Europe. AMO phase indicated in next to vertical axis.

In examining densities with respect to AMO fluctuations, the AIC of models one and two did not differ significantly (3795 and 3791 respectively, *p>*0.9). Therefore, the random intercept model was deemed the most appropriate, as it assumed that the strength of the relationship between density and the other variables changed randomly between years and latitudes (thus accounting for spatial and temporal variation of density reports). There was a negative correlation between density and the AMO index (*p*<0.001): in positive, warm phase years, cockle densities were lower (Fig 6). The sampling season also had an impact on the density records (*p*<0.01). In the summer, spring and autumn, the densities recorded were significantly lower (*p*<0.01 for each, Fig 5).

### 3.4. Historic reproduction patterns (macro level analysis)

Following the application of exclusion criteria, 20 records of cockle spawning times were obtained. These ranged in latitude from Trondheim in Norway (63° N, 10° E; [56]) to the Ria de Vigo, Spain (42° N, 8° W; [57]). The first record of spawning was in 1954 in the UK [58]. Few records were obtained from the 1950s to the 1960s (Fig 6). However, spawning duration appeared to vary greatly, with the longest spawning periods recorded from the 1980s onwards (Fig 6). Spawning was observed for most of the year in the case of cockles in the French Channel in 1987 [59], when the AMO Index was in a cold phase. One of the shortest spawning periods was noted in the northernmost record, with Trondheim cockles only spawning during the month of July in 1967 [56], during an AMO warm phase. A month-long spawning was also observed in south Wales in 1958 [60]. Periods of spawning also varied within sites [61]. For example, in south Wales complete spawning occured in spring and summer 1982. However, following a severe winter in the next year, partial repetitive spawning occurred between summer and autumn.

### 3.5. Case studies (micro level analysis)

Three case study sites were chosen from the northern, central and southern latitudes of the main distribution of cockles. Dundalk Bay (53°57’N 6°18'W), the Bay of Somme (50°13’N 1°35'E) and the Ria de Arousa (42°35’N 8°52'W) were each chosen due to their importance as regional fisheries. The following factors were examined: fishing types, conservation, management, legislation, parasites, mass mortality and weather. Comparable data was not always available for each site (S4 Table).

#### 3.5.1. Dundalk Bay, Ireland

Dundalk Bay, located on the east coast of Ireland, has 44.5 km^2^ of fished cockle beds. In the 1970s, infrequent documents of landings from Dundalk Bay emerged [62], with hydraulic suction and non-suction dredging, as well as hand gathering, being used in the fishery [62,63]. Dundalk Bay has been the dominant source of cockles in Ireland since 2001 [64].

As a Special Area of Conservation (SAC) and a Special Protected Area (SPA), the bay is subject to an assessment, prior to the opening of the fishery [65]. Regulations (SI No 532) and a Total Allowable Catch (TAC) were first introduced in 2007. Subsequently, additional, more formal, legislation was introduced in 2011 as a result of the Fisheries Natura Plan, required under the EU Habitats Directive (HD, 92/43/EEC; [65]). The annual TAC was set according to the stock biomass, which was assessed prior to the fishery annually. This legislation also outlined details on minimum landing size and gear specifications (Table 1). In 2007, approximately 668 tonnes of cockles were commercially harvested, lower than the agreed 950 tonne TAC [64]. A TAC of zero was enforced in 2008, 2010, 2013 and 2014 due to small size or low density [65]. A second five-year Fishery Natura Plan is in place since 2016. Its principle aim is to ensure protection of the habitats within Dundalk Bay, particularly to protect the cockle beds as a food source for oystercatchers (*Haematopus ostralegus*) [65].

**Table 1:** Key differences between three sites included in the case study of *Cerastoderma edule*.

	Dundalk (Ireland)	Somme (France)	Arousa (Spain)	Sources
First Legislation	2007	2013	1973	[65,67,77]
Mortalities	No Data	Yes (Eutrophication, Parasites)	Yes (Parasites)	[22,67,70]
Pathogens	No Data	*Vibrio aestuarianus*	*Marteilia*	[22,70]
Average Temperature	9.55°C	10.31°C	13.79°C	[42–44]
Maximum Harvest	668 tonnes (2007)	20,000 tonnes (1913)	2671 tonnes (2008)	[65,69,77]
Area Harvested	44.5km^2^	72 km^2^	†230km^2^	[62,67,73]
Max. Mean Density (ind/m^2^)	36 (2009–2010)	27.74 (2014)	527 (1997–2004)	[67,78,79]
Max. Mean Biomass (g/m^2^)	134.8 (2008)	34.62 (2014)	No Data	[67,78]

† Not all of this area is covered in cockle beds

In Dundalk Bay, no mass mortalities have been observed and no studies have detailed the presence of parasites and disease in the area. However, a number of parasites were observed in the nearby Dublin Bay in 2005, including digeneans, which have been implicated in mortalities elsewhere in Europe [20].

**Fig 7:**
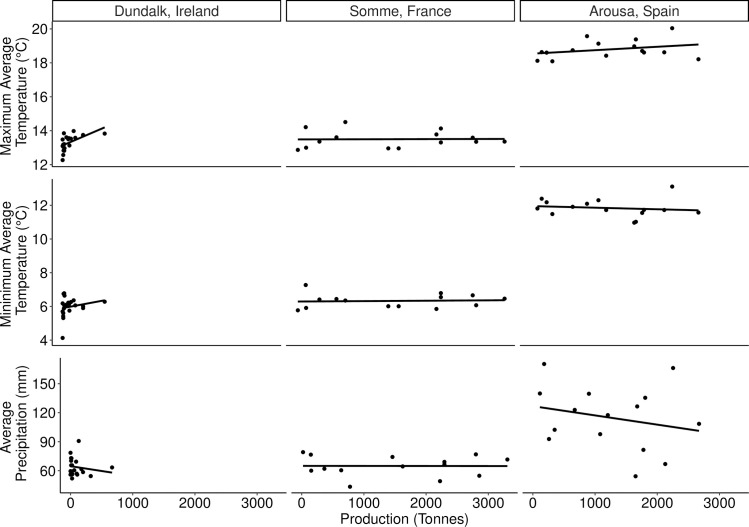
Relationships between climatic variables (minimum and maximum average temperature and annual average precipitation) and cockle production in the three key production areas in Ireland, France and Spain. Climate data was sourced from nearby weather stations [42–44].

Records show that climatic changes are influencing the health and biomass of cockles in Ireland. A severe cold spell was experienced in 2010 [66]. The entire year was generally cooler, with a mean temperature of 8.2°C, compared with the average of 9.6°C from 1942 to 2018 [42]. This coincided with lower precipitation and a decrease in cockle biomass (Fig 7). In general, years with higher minimum average temperatures, and years with lower precipitation, resulted in larger cockle harvests in Dundalk (Fig 7). However, an exception to this was noted in 2010. The highest biomass (and density) were observed in 2006, a year which was, on average, warmer than any other year between 2003 and 2011 [42].

#### 3.5.2. Bay of Somme, France

The Bay of Somme estuary contains 72 km^2^ of intertidal habitat and is the largest estuary in northwest France [67]. Cockles are gathered here by professional shore-fishers [67]. There are some discrepancies between data from different sources in the Bay of Somme. An absence of capture information was reported by the FAO between 1950 and 1977, while other reports state that cockle harvesting was occurring during this time [68]. For 2010, Thomas [67] reported a higher volume of cockles harvested than was reported by the FAO [14] (2855 vs 1496 tonnes). Despite the differences in data, the Bay of Somme accounted for a large proportion of French cockle harvests, both now and historically [14,68,69].

The bay is provided with a number of protections as an SPA, Marine Protected Area, Nature Reserve and Ramsar Convention site [67]. The first available record of legislation in the Bay of Somme exists from 2013, where the minimum capture size was 30 mm for cockles, and the fishery opened traditionally from September to December, with 339 fishers active. Since 2017, the legal fishing size was reduced to 27 mm for professional fishers, followed by recreational fishers in 2019.

Mass mortalities were reported in the Bay of Somme from 1981 to 1990, as a result of eutrophication [68], and further mortalities were reported in 2012, 2015 and 2018 due to the pathogenic bacteria *Vibrio aestuarianus* [70]. While some parasitological surveys have been conducted in France, these are primarily towards the south of the country and may not be representative [71,72]. The landings in the Bay of Somme did not appear to be influenced by any of the three examined variables.

#### 3.5.3. Ria de Arousa, Spain

Ria de Arousa is one of the four largest estuaries in Galicia, Spain, covering an area of 230 km^2^ [73]. It supported an MSC certified cockle fishery from 2013 to 2018 [74]. Cockles here are fished by hand and boat, while recreational fishing is not permitted [75]. Three types of exploitation are allowed under the management regime of Galicia. Two are territorial concessions, which are leased to a fishers’ guilds. There are also free access areas, which are directly managed by the government and require a license to operate in [75].

As the Ria de Arousa is a *Marteilia* impacted area, the free access fishery has a lower daily quota in comparison with other fisheries in Galicia. By 2018, cockle production had yet to recover following a *Marteilia* related mass mortality in 2012 [22]. This is unlike the recovery that followed a mortality in 2006, which occurred after the end of the fishing season, the highest year of production for the Ria de Arousa on record [22].

No apparent relationship was observed between the environmental variables (minimum/maximum average monthly temperature or precipitation. However, decreased salinity was implicated in the mortality event of 2006, as a result of increased precipitation [76].

#### 3.5.4. Comparison between the three sites

Comparable data was not always available from each site (Table 1). Mortality events were observed in both the Bay of Somme and Ria de Arousa, however of the three sites, only the Ria de Arousa was surveyed for parasites and pathogens [22]. A key difference evident between the sites was climate. Predictably, average temperatures were lowest in Dundalk and increased towards lower latitudes (Table 1). However, the increased temperatures did not result in obvious differences between cockle densities. Decreased precipitation (and increased salinity), lead to reductions in biomass in Dundalk. Conversely, increased precipitation and decreased salinity lead to reduced production in Ria de Arousa (Fig 7).

## 4. Discussion

The gathering of historical data can be a difficult endeavour for several reasons, as observed in this study: data gaps, complexity, or incomparable data collected for other purposes. This analysis of secondary data provides a holistic overview of cockle populations, however it is very difficult to examine detailed trends due to the complexity of derived datasets. A large amount of research was reactive, focussing on issues impacting fisheries at a particular time, e.g. mortality causes or recruitment issues. Data collected to-date on cockle populations has often been sporadic, disjointed and lacking in intra- and cross-border coordination. Studies focussed on one or only a few parameters and, understandably, were not carried out with the vision of future analysis. These findings of gaps in the dissemination of knowledge highlights the gaps that also exist between the research and practice communities.

It is important to evaluate the rationale used in this study. In terms of “the knowledge”, a large volume of secondary data was obtained detailing cockle populations in the past. This knowledge however was limited in space and time, with some areas/times with greater detail than others. In terms of “the source”, the scientific community was at the centre, with a large quantity of this presumably aimed at an industry or scientific “audience”. At a macro level, it appeared that knowledge mobilisation was unsuccessful and this was further evidenced in the lack of choices for case study locations. At the micro level, which examined important areas for cockle fisheries, it was evident that knowledge mobilisation was sometimes happening at a local level, resulting in successful fisheries monitoring translating to management plans.

Many of the sources of data included in this study, such as newspaper articles are generally not considered in scientific studies [80] (Fig 1), despite such resources being accessible to a larger audience, across a range of disciplines and sectors. However, in this well studied species it was evident that a research-practice gap exists. Very little information was derived from conference proceedings or workshops, highlighting the lack of active engagement between all relevant stakeholders. This study was larger in geographical scope than other marine historical ecology studies [34, 81], which brings another issue to the fore. Lack of cross border communication was evident with most studies only dealing with local issues, rather than a comparison across the range of the species. This highlights the requirement to improve knowledge mobilisation not just among local audiences, but across borders. However, the examination of the case studies highlights the benefit of bridging the gap between research and practice, as scientific monitoring in all three case study sites was shown to feed the management and policies there.

In many cases examined by this study, important factors such as gear type and sampling effort were not reported. Furthermore, sampling schemes (e.g. transect vs quadrats) varied between studies. It has previously been reported that sampling schemes can impact density estimates [82]. However, even when these confounding effects are not taken into account, estimates in changes of cockle abundance may still be determined [32], although should be considered cautiously when examining trends in species. A common approach to sampling, using similar equipment, time periods and reporting, would provide more informed robust data for decision making.

Typically, marine historical ecology is conducted not just through internet and electronic methods (as in this study), but through methods including physical searches and interviews [33]. Only using electronic means would be beneficial, reducing the costs, time and effort related to field work, particularly when examining the entire range of a species. When gathering data for this study, a lack of accessible data was discovered in the public realm. It would be more worthwhile to conduct more local scale case studies, and acquiring physical archival information and local knowledge, which would provide a more determinate overview of the factors impacting cockles. Such case studies would have more in-depth information than reported here and could be pooled to provide a holistic overview of fisheries. However, it must be noted that such a project would require greater financial investment.

In cockles, the study species, it appeared that climate had an impact on a global scale, with an overall increase in density in years with a negative AMO (“cooling” trend, Fig 1). There is currently an emerging trend of a negative AMO index in the northern Atlantic [83], which may lead to cooler sea surface temperatures and thus be beneficial to cockle populations. However, when examining local trends (micro level) using historical data, deriving a trend is much more difficult.

While it is known that environmental factors can play a major role in influencing cockle populations [17,18,84], it was found that management regimes and legislation also impact cockles at a regional level. It was evident that fishing methods vary across the range of cockles (S4 Table), some of which are more destructive than others (e.g. dredging vs hand picking). Conversely, management and legislation can have a positive impact on cockle densities and biomass. This was particularly evident in Dundalk, where a TAC was implemented (Table 1) [65]. This measure prevented uncontrolled fishing, even when biomass was very high, facilitating improved recruitment and population expansion. Acquiring information on legislation detailing fishing techniques and management regimes for historic populations was difficult, thus making it difficult to properly examine trends at specific fisheries.

In spite of some of the limitations, a number of unexpected findings were encountered during this study. Distribution of *C*. *edule* was well known along Atlantic coasts of European countries, evident of the established fisheries (Fig 3). However, modern reports of *C*. *edule* were recorded in the Mediterranean (Fig 3), despite the lack of reported fisheries in this region [14]. This is a difficulty encountered in other studies, where taxonomic issues have led to errors [85]. While it is possible that this was due to difficulty in differentiating *C*. *edule* from *C*. *glaucum*, this is an interesting finding that is undergoing investigation [86]. In addition, the findings concur with previous literature stating that densities of cockles tend to be higher towards higher latitudes (Fig 4) [25]. While Senegal (the southernmost record of cockles) has previously reported cockle fisheries [14], the dearth of reports from southern Morocco to Senegal (Fig 3) may be an indicator of low densities of cockles in this region [13], lack of funding for this research topic, or low interest in this fishery. Such findings highlight the advantages to carrying out secondary data analyses.

A number of further recommendations can be made to improve knowledge mobilisation in commercially important species like cockles. Searches in this study were conducted via electronic means and with other scientific researchers. However, collaborating with stakeholders, including fishers and managers would allow for access to “closed knowledge sharing networks” [3]. Open science is also a vital factor in examining the impacts of climate on species [87]. It is essential that information is open and easily sharable, particulary in the case of an important resource [1]. This study highlights the necessity of a “one stop” online resource where those gathering data (regardless of their community), can digitise new and pre existing information. Incentives for the sharing of data are widely lacking [2]. Such incentives should be provided (as demonstrated by the COCKLES Project [88]) to facilitate easier sharing and access to data. Further incentives to promote the impact of collaborations should be encouraged through publishing this impact or including a chapter in dissertations [2,89].

The knowledge and information compiled in this study offers an insight to knowledge mobilisation in commercially fished species. While the variability of cockle populations is affirmed in this study, a more in-depth analysis was not achievable due to the lack of access to concise data. Nonetheless, the knowledge and information compiled offers many opportunities for improved, evidence-based, fisheries management and conservation. It was apparent that, previously, studies were not conducted with the intention of large-scale comparisons. To allow for more accurate predictions in the future, it would be worthwhile to employ a standardised protocol for surveying cockle populations and create a common web portal for these results. Simplifying the delivery channel will enable a more reliable relay of cockle data (i.e. knowledge) to a broader audience of stakeholders, allowing more consistent reporting of results. These recommendations are necessary for fisheries management, conservation and science to support the future sustainability of not only this industry, but other fish and shellfish species, that many European coastal communities depend on for their incomes and culture.

## Supporting information

S1 Table Historical topics. Classification of topics in each data source in a study of historic populations of *Cerastoderma edule*.
(DOCX)

S2 Table Details of mixed effects model. Variables, variable types and factor levels included in the mixed effects model examining density in cockles (*Cerastoderma edule*).
(DOCX)

S3 Table Mixed effects models. Models examined by linear mixed effects model fit by REML, on the dependent variable, density.
(DOCX)

S4 Table Cockle harvesting details. Summary of available information on harvest and legislation relating to cockles across Europe.
(DOCX)

S5 Table Results of mixed effects models. Output of a the final linear mixed effects models examining variations in cockle density.
(DOCX)

S1 Fig Assumptions for mixed effects model. Assumptions for random intercept and slope model examining trends in density of cockles.
(DOCX)
