# Supplementary material for: Mobilisation of data to stakeholder communities. Bridging the research-practice gap using a commercial shellfish species model
Source: PLoS One. 2020 Sep 23;15(9):e0238446. doi: 10.1371/journal.pone.0238446 (PMC7510983; doi:10.1371/journal.pone.0238446)
Supplement: S1 Table — Classification of topics in each data source in a study of historic populations of Cerastoderma edule. (DOCX) [file pone.0238446.s001.docx]

| Topic | Content type |
| --- | --- |
| Cockles | Relating to the biology of cockles (morphology, reproduction, growth, abundance etc.) |
| Cultural | Aspects of cockles related to human culture and society (e.g. food, art) |
| Ecosystem | Dealing with interactions between cockles and other species or the ecosystem (e.g. sediment dynamics, food for birds). |
| Fishery | Relating to landings, fishing types, legislation |
| Parasites | Details of parasitology (and lesions, disseminated neoplasia) in cockles |
