# Supplementary material for: Mobilisation of data to stakeholder communities. Bridging the research-practice gap using a commercial shellfish species model
Source: PLoS One. 2020 Sep 23;15(9):e0238446. doi: 10.1371/journal.pone.0238446 (PMC7510983; doi:10.1371/journal.pone.0238446)
Supplement: S2 Table — Variables, variable types and factor levels included in the mixed effects model examining density in cockles (Cerastoderma edule). (DOCX) [file pone.0238446.s002.docx]

| Variable | Variable Type | Factor Levels | Comments |
| --- | --- | --- | --- |
| AMO Index | Continuous | - | - |
| Season | Discrete | All Year  Autumn  Autumn, Winter  Not Stated  Spring  Spring, Summer  Summer  Winter | An observation occurred “All Year”, if the density measurement was a result of sampling in three or more seasons.  Autumn, Winter/Spring, Summer, indicates that an observation was recorded over two seasons |
| Sampling Type | Discrete | Intensive  Extensive | Levels were chosen to reflect the impact of the sampling gear in a commercial setting. Extensive sampling was obtained by hand gathering (e.g. quadrats, raking, transects). Intensive samples were obtained by dredging, cores and grabs. |
| Latitude | Continuous | - | - |
| Age | Discrete | Adult  Juvenile  Both  Not Stated | - |
