# Supplementary material for: Mobilisation of data to stakeholder communities. Bridging the research-practice gap using a commercial shellfish species model
Source: PLoS One. 2020 Sep 23;15(9):e0238446. doi: 10.1371/journal.pone.0238446 (PMC7510983; doi:10.1371/journal.pone.0238446)
Supplement: S3 Table — Models examined by linear mixed effects model fit by REML, on the dependent variable, density. (DOCX) [file pone.0238446.s003.docx]

| **Model** | **Fixed Effects** | **Random Effects** | **AIC** |
| --- | --- | --- | --- |
| 1 | AMO, Sampling Type, Season, Latitude, Year, Age | Year\|Latitude | 3794 |
| 2 | AMO, Sampling Type, Season, Latitude, Year, Age | 1\|Latitude | 3791 |
| 3 | AMO, Sampling Type, Season, Latitude, Year, Age | 1\|Year | 3813 |
