# Supplementary material for: Mobilisation of data to stakeholder communities. Bridging the research-practice gap using a commercial shellfish species model
Source: PLoS One. 2020 Sep 23;15(9):e0238446. doi: 10.1371/journal.pone.0238446 (PMC7510983; doi:10.1371/journal.pone.0238446)
Supplement: S4 Table — Summary of available information on harvest and legislation relating to cockles across Europe. (DOCX) [file pone.0238446.s004.docx]

|  | **Denmark** | **France** | **Germany** | **Ireland** | **The Netherlands** | **Portugal** | **Spain (Galicia)** | **The UK** |
| --- | --- | --- | --- | --- | --- | --- | --- | --- |
| Maximum Capture (Tonnes) | 7,699 | 40,000 | 8,133 | 668 | 80,000 | 4,714 | 21,500 | 46,615 |
| Year of Max Capture | 2015 | 1913 | 1983 | 2007 | 1989 | 2017 | 1968 | 1991 |
| Number of Key Production Areas | 2+ | 3+ | 1+ | 1+ | 2+ | 3+ | 44+ | 12+ |
| Year of First Legislation | ? | 1990 | 1989 | 2007 | 1999 | 1987 | ca. 1973 | ? |
| Gear Types | Dredging, Hand gathering | Hand gathering, Rakes | Hand gathering | Hydraulic suction dredge, Hand gathering | Hand gathering, Rakes, Hydraulic dredge | Rake, Harvesting knife | Grubber hoe, Hand rake, Bullrake, Hand dredge | Hand rake, Trailed pump scoop dredge |
| Fisheries in Protected Areas | ? | Banc d'Arguin, Bay of Saint Brieuc,  Bay of Somme,  Bay of Authie | ? | Dundalk, Tramore, Castlemaine | ? | Ria de Aveiro, Ria Formosa | Ria de Arousa  Ria de Pontevedra  Ria de Vigo | Burry Inlet, Morecambe Bay, Solway Firth, Thames Estuary |
| TACs | Limfjord | No but quotas per fisher and bed | ? | Dundalk | Wadden Sea | No | No but yearly management plans after stock assessment | Burry Inlet, Solway Firth, Thames Estuary |
| Minimum Capture Size | ? | 27-30 mm | ? | 17-22 mm | 21 mm | 25 mm | 25-28 mm | 10-23.8 mm |
| ? No data available |  |  |  |  |  |  |  |  |
