# Supplementary material for: Mobilisation of data to stakeholder communities. Bridging the research-practice gap using a commercial shellfish species model
Source: PLoS One. 2020 Sep 23;15(9):e0238446. doi: 10.1371/journal.pone.0238446 (PMC7510983; doi:10.1371/journal.pone.0238446)
Supplement: S5 Table — Output of a the final linear mixed effects models examining variations in cockle density. (DOCX) [file pone.0238446.s005.docx]

| Variable | Factor Level | DF | t-value | *p* |
| --- | --- | --- | --- | --- |
| AMO | - | 205 | -3.972708 | 0.0001 |
| Season | Autumn | 205 | -3.410272 | 0.0008 |
|  | Autumn, Winter | 141 | -0.791095 | 0.0754 |
|  | Not stated | 205 | 0.622367 | 0.5344 |
|  | Spring | 205 | -2.407012 | 0.0170 |
|  | Spring, Summer | 205 | -0.785518 | 0.4331 |
|  | Summer | 205 | -3.523285 | 0.0005 |
|  | Winter | 205 | -1.735372 | 0.0842 |
|  | Winter, Spring | 205 | -1.702027 | 0.0903 |
