# Supplementary figures and images for: Mobilisation of data to stakeholder communities. Bridging the research-practice gap using a commercial shellfish species model
Source: PLoS One. 2020 Sep 23;15(9):e0238446. doi: 10.1371/journal.pone.0238446 (PMC7510983; doi:10.1371/journal.pone.0238446)

| 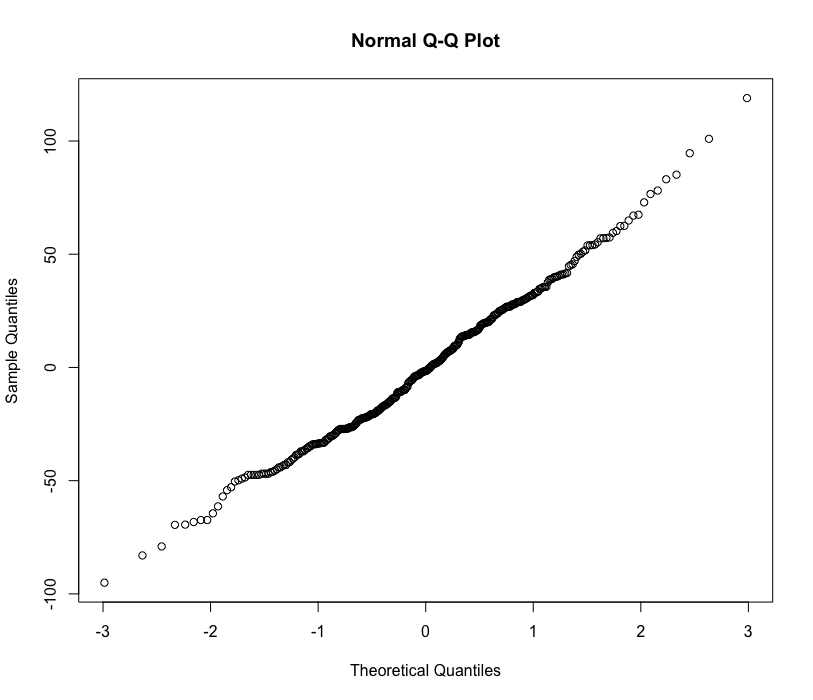 | 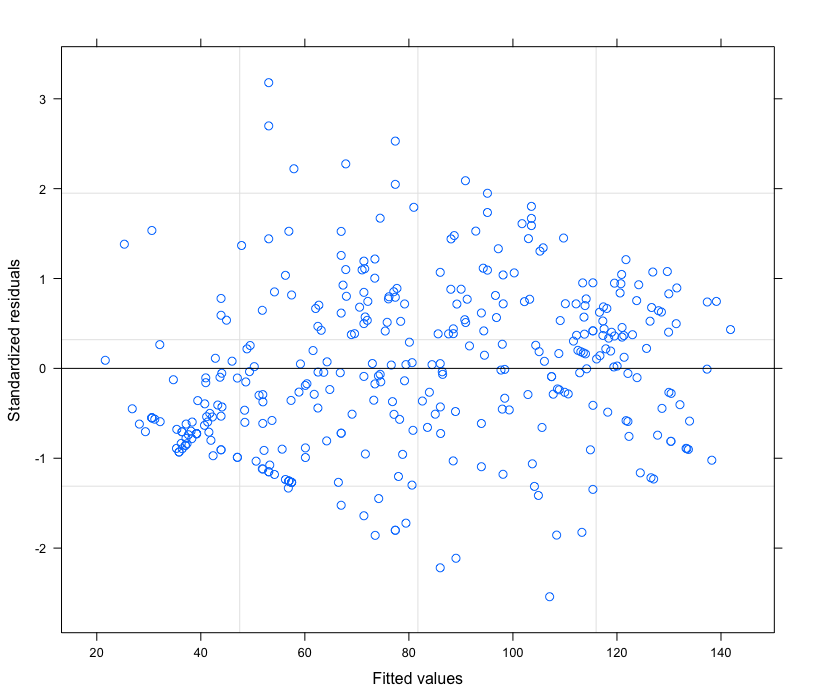 |
| --- | --- |
| 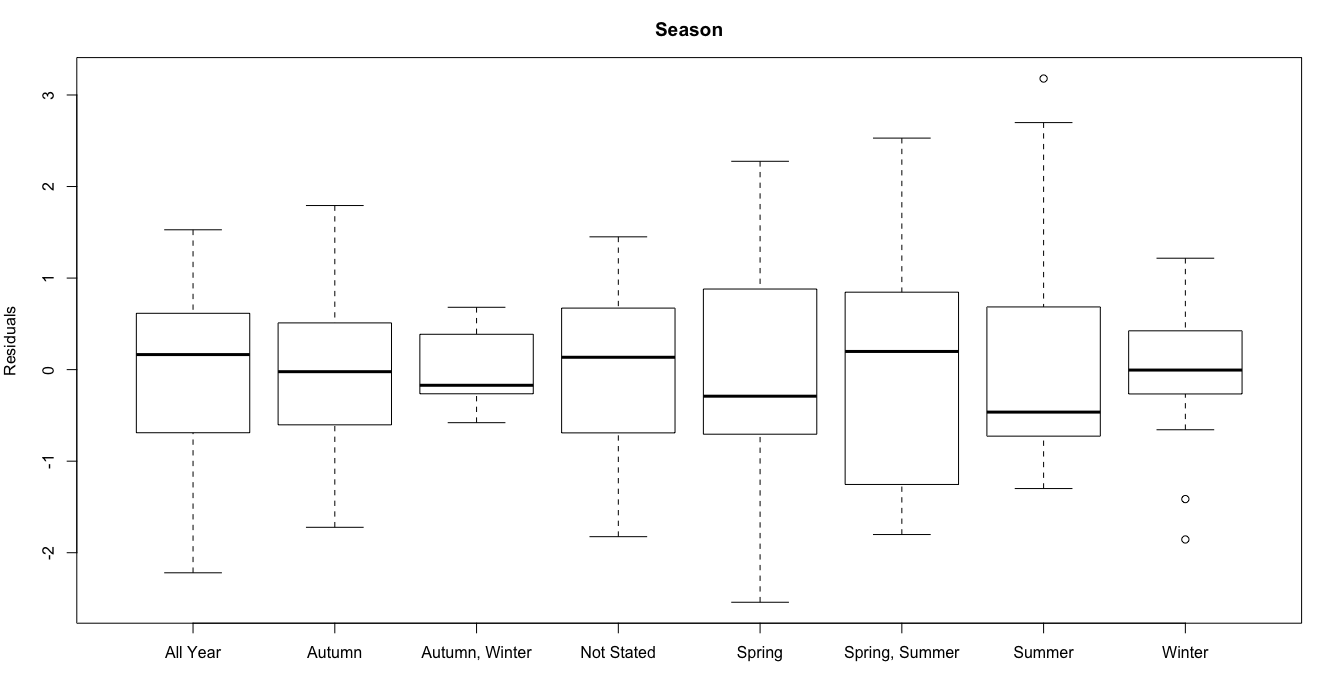 | 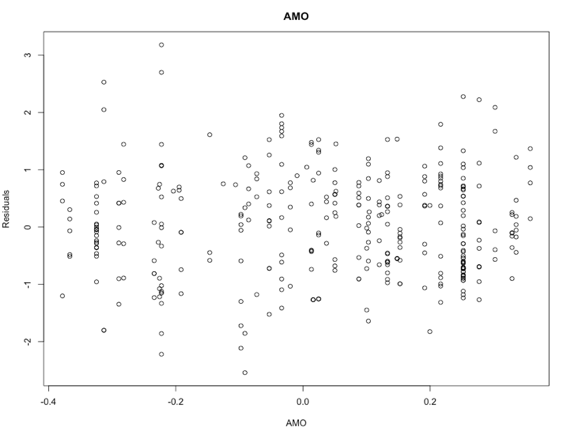 |

Supplement: S1 Fig — Assumptions for random intercept and slope model examining trends in density of cockles. (DOCX) [file pone.0238446.s006.docx]
